# Human immunodeficiency virus (HIV) among men who have sex with men: results of the first integrated biological and behavioral survey in Burkina Faso, West Africa

**DOI:** 10.1186/s12889-018-6361-1

**Published:** 2019-01-03

**Authors:** Henri Gautier Ouedraogo, Odette Ky-Zerbo, Ashley Grosso, Sara Goodman, Benoît Cesaire Samadoulougou, Grissoum Tarnagda, Adama Baguiya, Simon Tiendrebeogo, Marcel Lougue, Nongoba Sawadogo, Yves Traore, Nicolas Barro, Stefan Baral, Seni Kouanda

**Affiliations:** 1 Biomedical Research Laboratory, Biomedical and Public Health Department, Institut de Recherche en Sciences de la Santé (IRSS), 03BP7192, Ouagadougou, West-Africa Burkina Faso; 2 University Ouaga 1 Joseph Ki-Zerbo, Ouagadougou, Burkina Faso; 3 Institut Africain de Santé Publique, Ouagadougou, Burkina Faso; 4 Programme d’Appui au Monde Associatif et Communautaire, Ouagadougou, Burkina Faso; 5 Department of Epidemiology, Johns Hopkins Bloomberg School of Public Health, Baltimore, MD USA; 6 Centre Hospitalier Regional de Kaya, Kaya, Burkina Faso

**Keywords:** HIV, Epidemiology, Infection, MSM, Burkina Faso, West Africa

## Abstract

**Background:**

Many men who have sex with men (MSM) are at significant risk for HIV infection. The objective of this study was to determine the prevalence and correlates of HIV infection among MSM in Burkina Faso.

**Methods:**

A cross-sectional biological and behavioral survey was conducted from January to August 2013 among MSM in Ouagadougou and Bobo-Dioulasso. MSM 18 years old and above were recruited using respondent driven sampling (RDS). A survey was administered to study participants followed by HIV testing. Population prevalence estimates and 95% confidence intervals (CI) adjusted for the RDS design were produced using the RDS Analysis Tool version 6.0.1 (RDS, Inc., Ithaca, NY).

**Results:**

A total of 662 MSM were enrolled in Ouagadougou (*n* = 333) and Bobo-Dioulasso (*n* = 329). The majority were unmarried, with an average age of 22.1 ± 4.4 years old in Ouagadougou and 23.1 ± 4.7 years old in Bobo-Dioulasso. RDS-adjusted HIV prevalence was 1.7% (95% CI: 0.9–3.1) in Ouagadougou and 2.7% (95% CI: 1.6–4.6) in Bobo-Dioulasso. HIV prevalence among MSM under 25 years old was 1.3% (95% CI: 0.6–2.8) and 0.9% (95% CI: 0.4–2.5) respectively in Ouagadougou and Bobo-Dioulasso, compared to 5.4% (95% CI: 2.2–12.5) and 6.6% (95% CI: 3.4–12.3) among those 25 years old or older in these cities (*p* = 0.010 and *p* < 0.001).

**Conclusions:**

Results from this first biological and behavioral survey among MSM in Burkina Faso suggest a need for programs to raise awareness among MSM and promote safer sex, particularly for young MSM to prevent HIV transmission. These programs would need support from donors for innovative actions such as promoting and providing pre-exposure prophylaxis, condoms and water-based lubricants, HIV counseling, testing, early treatment initiation and effective involvement of the MSM communities.

## Background

Globally, Human Immunodeficiency Virus (HIV) infection remains a significant issue for key populations such as men who have sex with men (MSM) [1–3]. Although the first HIV cases were among MSM [4], the burden of HIV transmission in this group has been ignored by HIV control programs for many years in several African countries like Zambia, Uganda, Mauritania, Cameroon, Chad, Mali, and Burkina Faso [5]. Most programs focused on the prevention of heterosexual and mother to child transmission to the detriment of sexual transmission between men, until several studies conducted during the last decade confirmed the high prevalence of HIV among MSM [6–11]. There is no specific law that criminalizes same-sex sexual behaviors in Burkina Faso, but sex between men is stigmatized [12]. It is often associated with a hidden HIV epidemic in Africa [13, 14], which underscores the progressive reorientation of including MSM as a target population for HIV prevention and care [5].

Indeed, epidemiological studies conducted in Sub-Saharan Africa have highlighted an extreme vulnerability to sexually transmitted infections (STIs) among MSM, a prevalence of unsafe sexual practices and a higher rate of HIV infection than in the general population [15–18]. In West Africa, HIV prevalence among MSM fluctuates across and within countries. It was reported to be 4.7 and 34.3% in Ghana [19], 18% in Cote d’Ivoire [20]. In Nigeria, HIV among MSM in Abuja, Ibadan, and Lagos were 34.9, 11.3, and 15.2%, respectively [21].

The estimated HIV prevalence among the general adult male population in Burkina Faso is 0.8% [22]. However, little is known about HIV prevalence among MSM [8]. Data are needed on HIV prevalence and behaviors among MSM for a better control of the epidemic.

In order to fill this gap, and provide evidence for HIV programs, we conducted this study to estimate HIV prevalence among MSM in the two largest cities of Burkina Faso (Ouagadougou and Bobo-Dioulasso). This was first study among MSM in Burkina Faso that included both a survey and HIV testing.

## Methods

### Study design

This was a cross-sectional survey among MSM using respondent driven sampling (RDS). RDS is a peer-recruitment sampling method designed to collect rigorous, representative data from hard-to-reach populations [23–25]. In preparation for this first integrated biological and behavioral survey, formative pre-survey research included formal meetings in each site with MSM, local organizations, and government officials to explore MSM’s willingness to recruit their peers, challenges in finding diverse segments of this hidden population, and preferences expressed by MSM for all study procedures.

### Setting

The study took place in Burkina Faso’s two largest cities: Ouagadougou (the capital) in the Centre and Bobo-Dioulasso in the West.

### Study population and recruitment

MSM were eligible to participate if they were (i) at least 18 years old, (ii) assigned male sex at birth, (iii) reported they had anal sex with a man at least once in the past 12 months, (iv) were able to provide informed consent in French, Mòoré, or Dioula, (v) had a valid RDS coupon, (vi) lived in either Ouagadougou or Bobo-Dioulasso for at least the past three months, and (vii) agreed to complete a survey and HIV testing.

Six MSM seeds in Ouagadougou and four MSM seeds in Bobo-Dioulasso were purposely selected to initiate recruitment chains. We chose seeds who met the study eligibility criteria, represented diverse demographics (age, education, marital status, language, and HIV status), and who were willing to promote the study. Three seeds of 45, 30 and 23 years old started in Ouagadougou and failed to recruit.

**Fig. 1:**
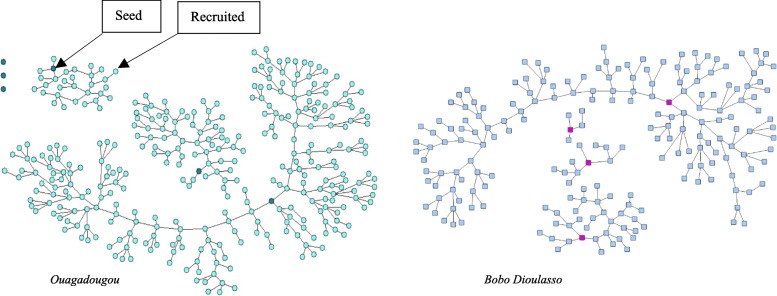
Respondent driven sampling recruitment chains of MSM in Ouagadougou and Bobo-Dioulasso, Burkina Faso

After giving informed consent, seeds were required to complete a survey and have their blood drawn for HIV testing. These seeds were each provided with three coded coupons, which were valid for four weeks, to recruit peer MSM from their social networks. Individuals who were recruited by seeds and enrolled in the study were then provided with three coded study coupons for further recruitment of peers. This process continued until the target sample size was reached in each city. To assess whether convergence of key characteristics was achieved [26], we examined convergence plots of age, educational status and sexual orientation, that we anticipated to could be associated with network structure and HIV infection. As we approach the sample size in each city, the number of coupons was reduced from 3 to 2. The last wave of study participants did not receive a coupon to recruit their peers. The maximum numbers of sample waves per site were 15 in Ouagadougou and 16 in Bobo-Dioulasso. Figure 1 presents RDS recruitment chains of MSM in Ouagadougou and Bobo-Dioulasso.

Participants received male condoms, condom-compatible lubricants, HIV education materials, and information regarding existing services. They also received 2000 West African Communauté Financière Africaine franc (XOF, ~ $4 United States dollars [USD]) for their time and transportation costs for each study visit and 1500 XOF (~ $3 USD) per successfully eligible peer recruited to be part of the study (for up to three peers). To avoid individuals participating multiple times, a single survey office was used in each study site. At each site, trained staff included a site manager, a coupon manager, two data collectors, an HIV test counselor, and a lab technician. Each of them was trained to avoid multiple participations through facial and physical recognition.

### Sample size

The recruitment framework entailed 345 MSM in each city (Ouagadougou and Bobo Dioulasso). Sample size calculations were based on the assumption that populations who always use condoms have a 75% lower HIV prevalence than populations who do not, and the effectiveness of condoms is roughly 80%, with 73% used as a conservative estimate [27]. Overall, HIV prevalence was assumed to be 15%, with a 19% prevalence among those who did not consistently use condoms. A design effect of 1.5 associated with RDS, a significance level of 0.05 and a power of 80% were employed. Condom use was used to calculate sample size because we were interested in using the study data as a baseline for behavior change.

### Data collection

Data were collected from January to April 2013 in Ouagadougou and May to August 2013 in Bobo-Dioulasso. Following written informed consent, MSM participants completed interviewer-administered face-to-face surveys in a private room. Topics included participants’ socio-demographic characteristics, concurrence sexual partnerships and sexual behaviors during the last 12 months (with men and women), condom use during the last 12 months and the last sex with regular or casual male and female sexual partners, knowledge and practices related to STIs and HIV. Information on concurrent sexual partners was obtained by asking participants if in the last 12 months, there was any time when they had two or more regular sexual partners (males or females) at the same time.

### Laboratory method for HIV testing

Following completion of the survey, HIV counseling and testing, based on the Burkina Faso official guidelines, were conducted for all participants. A trained nurse and lab technician from Institut de Recherche en Sciences de la Santé (IRSS, Ouagadougou) respectively conducted pre- and post-test counseling and venous blood specimen collection (~ 5 ml) and HIV testing. The first step was to perform a rapid test using Alere Determine™ HIV-1/2 kit (Alere, Inc., Waltham, Massachusetts). This was followed by ImmunoComb® II HIV 1&2 BiSpot kit (Orgenics Ltd., Israël) as a second test for differential detection of antibodies to HIV types 1 and 2, only if the first test was positive. Any discordant results were tested using the ImmunoComb II HIV 1&2 CombFirm kit. (Orgenics Ltd., Israël) positive or negative status. Participant codes were used to link results of the surveys with test results as well as facilitate the provision of test results and appropriate treatment or referrals. Participants who chose to receive their results could do so on-site shortly after testing.

### Data processing

Data were entered using EpiData 3.1 (The EpiData Association, Odense, Denmark) and exported into Stata 14 (StataCorp, College Station, TX) for analysis. RDS original seeds were included for the analysis. For each city, population prevalence estimates and 95% confidence intervals (CI) adjusted for the RDS design were conducted using the RDS Analysis Tools (RDSAT) version 6.0.1 (RDS, Inc., Ithaca, NY). We present proportions separately for each city because the RDS networks were separate. RDS adjustment takes into consideration the probability of each participant to be included in the study. This probability was measured through weighting based on the size of each participant’s network. Network size was determined using the survey question: “How many different people do you know personally who are men who have sex with men? i.e., you know them and they know you, you have seen them in the last 2 years, and you could contact them if you needed to?” The mean network size was 17 in Ouagadougou (range: 1 to 600) and 21 in Bobo-Dioulasso (range: 1 to 150). Bivariate logistic regression analyses were conducted using Stata to assess correlates of testing positive for HIV in each city. RDS weights were included in the logistic regression analyses for each city. Due to low number of HIV positives, multivariate analysis was not performed.

### Ethical considerations and protection of the participants

The study received ethical approval from the Johns Hopkins Bloomberg School of Public Health Institutional Review Board and the Ethics Committee for Health Research (Comité d’éthique pour la recherche en santé, CERS) of Burkina Faso. Procedures were put in place to protect participants against risks. Surveys were conducted in a private setting. To minimize physical risks, collection of blood samples were performed by trained staff. Psychological risks for study staff that work first time with MSM were minimized by providing research ethics training and sensitivity for all staff on the study objectives and the specific needs of MSM. Confidentiality was maintained by using a unique study identifier rather than names on surveys, protecting all electronic data with passwords, and storing hard copies of data in locked cabinets. Participants who tested positive for HIV were referred to an appropriate HIV treatment center.

## Results

A total of 333 MSM in Ouagadougou and 329 in Bobo-Dioulasso, including the original seeds, participated.

### Socio-demographic characteristics

The mean age was 22.1 ± 4.1 years in Ouagadougou and 23.1 ± 4.7 in Bobo-Dioulasso. Participants’ socio-demographic characteristics are presented in Table 1. The majority of study participants were under 25 years old (83.6, 95% CI: 79.2–87.2 in Ouagadougou and 75.5, 95% CI: 70.6–79.9 in Bobo-Dioulasso), and unmarried (94.6, 95% CI: 91.6–96.5 in Ouagadougou and 96.7, 95% CI: 94.2–98.1 in Bobo-Dioulasso). Most were born in Burkina Faso. Most participants in Ouagadougou (71.8, 95% CI: 66.8–76.4) and Bobo-Dioulasso (55.2, 95% CI: 49.8–65.0) were students or pupils, while workers represented 21.6% (95% CI: 17.5–26.3) in Ouagadougou and 38.3 (95% CI: 33.2–43.7) in Bobo-Dioulasso. In terms of sexual orientation, respectively 51.0% (95% CI: 45.6–56.4) and 56.0% (95% CI: 55.0–61.3) of MSM in Ouagadougou and Bobo-Dioulasso reported being gay/homosexual. Less than half of participants were bisexual (44.1, 95% CI: 38.9–49.5 in Ouagadougou and 39.2, 95% CI: 34.1–44.6 in Bobo-Dioulasso). A minority in Ouagadougou (2.1, 95% CI: 1.0–4.4) and Bobo-Dioulasso (3.9, 95% CI: 2.3–6.6) were heterosexual. In both cities combined, 154 MSM out of 658 (23.4%) reported previous STI testing during the last 12 months.

**Table 1:** Socio-demographic characteristics of study participants (men who have sex with men) by city

	Ouagadougou		Bobo-Dioulasso	
Variables	n (RDS-unadjusted %)	RDS-adjusted % (95% CI)	n (RDS-unadjusted %)	RDS-adjusted % (95% CI)
Current age (years)				
<=24	277 (83.2)	83.6 (79.2–87.2)	246 (74.8)	75.5 (70.6–79.9)
> = 25	56 (16.8)	16.4 (12.8–20.8)	83 (25.2)	24.5 (20.1–29.4)
Total	333		329	
Marital status (with a woman)				
Single	313 (94.3)	94.6 (91.6–96.5)	316 (96.3)	96.7 (94.2–98.1)
Other^a^	19 (5.7)	4.8 (3.0–7.7)	12 (3.7)	3.3 (1.9–5.8)
Total	332		328	
Country of birth				
Burkina Faso	276 (82.9)	82.9 (78.4–86.6)	275 (83.6)	83.7 (79.3–87.3)
Other countries	57 (17.1)	17.1 (13.4–21.6)	54 (16.4)	16.3 (12.7–27.0)
Total	333		329	
Childhood environment				
Urban	291 (91.5)	91.5 (87.9–94.1)	309 (93.9)	94.1 (91.0–96.2)
Rural	27 (8.5)	8.5 (5.9–12.1)	20 (6.1)	5.9 (3.8–9.0)
Total	318		329	
Highest educational level				
None or primary	25 (7.5)	7.5 (5.1–10.9)	39 (11.9)	11.1 (08.4–15.3)
Secondary	242 (72.7)	72.9 (67.9–77.4)	225 (68.4)	68.8 (63.5–73.6)
University	66 (19.8)	19.6 (15.7–24.2)	65 (19.8)	19.8 (15.8–24.5)
Total	333		329	
Occupation				
Student/pupil	238 (71.5)	71.8 (66.8–76.4)	179 (54.4)	55.2 (49.8–65.0)
Employed	73 (21.9)	21.6 (17.5–26.3)	129 (39.2)	38.3 (33.2–43.7)
Unemployed	22 (6.6)	6.6 (4.4–9.8)	21 (6.4)	6.5 (4.3–9.8)
Total	333		329	
Number of biological children				
0	308 (92.5)	92.8 (89.5–95.1)	304 (92.4)	93.1 (89.8–95.3)
> = 1	25 (7.5)	7.2 (4.9–10.5)	25 (7.6)	6.9 (4.7–12.0)
Total	333		329	
Gender identity				
Man	237 (71.4)	71.6 (66.5–76.2)	201 (61.1)	61.0 (55.5–66.1)
Woman	22 (6.6)	6.6 (4.4–9.8)	75 (22.8)	22.8 (18.6–27.7)
Intersex	73 (22.0)	21.8 (17.6–26.6)	53 (16.1)	16.3 (12.6–20.7)
Total	332		329	
Sexual orientation				
Gay/homosexual	170 (51.1)	51.0 (45.6–56.4)	184 (55.9)	56.0 (55.0–61.3)
Bisexual	147 (44.1)	44.1 (38.9–49.5)	129 (39.2)	39.2 (34.1–44.6)
Heterosexual	7 (2.1)	2.1 (1.0–4.4)	13 (4.0)	3.9 (2.3–6.6)
Transgender	9 (2.7)	2.7 (1.4–5.2)	3 (0.9)	0.9 (0.3–2.8)
Total	333		329	
Had insertive anal sex in the past 12 months				
No	19 (5.7)	5.7 (3.6–8.7)	66 (20.1)	19.7 (15.8–24.4)
Yes	314 (94.3)	94.3 (91.3–96.4)	263 (79.9)	83.0 (75.6–84.2)
Total	333		329	
Had receptive anal sex in the past 12 months				
No	113 (33.9)	34.0 (29.1–39.3)	118 (35.9)	36.1 (31.0–41.5)
Yes	220 (66.1)	66.0 (60.7–70.9)	211 (64.1)	63.9 (58.5–69.0).
Total	333		329	
Number of male anal sex partners during the last 12 months				
1	75 (22.6)	22.7 (18.4–27.5)	93 (28.4)	28.6 (23.9–33.7)
2–3	133 (40.1)	41.0 (34.9–45.5)	126 (38.4)	38.3 (33.1–43.7)
> = 4	124 (37.3)	37.2 (32.2–42.6)	109 (33.2)	33.1 (28.2–38.4)
Total	332		328	
At least 2 sexual partners currently				
No	60 (18.1)	17.7 (13.9–22.2)	76 (23.3)	23.3 (19.0–28.2)
Yes	272 (81.9)	8.3 (77.8–86.1)	250 (76.7)	76.7 (71.8–81.0)
Total	332		326	
Tested for STIs during the last 12 months				
No	242 (73.6)	73.8 (68.8–78.3)	262 (79.6)	79.9 (75.3–83.9)
Yes	87 (26.4)	26.2 (21.7–31.2)	67 (20.4)	21.0 (16.1–24.7)
Total	329		329	

^*^Married or cohabiting, divorced, separated

### Awareness of HIV transmission risks, sexual behaviors, and use of condoms

In Ouagadougou, less than half of MSM (46.7, 95% CI: 41.4–52.1) incorrectly believed that there is no difference between vaginal, anal or oral sex in terms of HIV transmission risk. However, 41.7% of MSM in Ouagadougou compared to 27.2% in Bobo-Dioulasso incorrectly believed that vaginal sex has a higher risk of HIV transmission than other types of sexual intercourse (Table 2). In addition, the majority of MSM in both cities (72.8% in Ouagadougou and 67.9% in Bobo-Dioulasso) incorrectly believed the risk of HIV acquisition is similar for insertive or receptive anal sex. Only 15.2% in Ouagadougou and 16.1% in Bobo-Dioulasso were aware that receptive anal intercourse carries higher risk of HIV acquisition than insertive sexual intercourse.

**Table 2:** Awareness of HIV transmission risks, gender of sexual partners, and condom use among men who have sex with men by city

Variables	Ouagadougou		Bobo-Dioulasso	
	n (RDS-unadjusted %)	RDS-adjusted % (95% CI)	n (RDS-unadjusted %)	RDS-adjusted % (95% CI)
Knowledge of the type of sex with the highest risk of HIV acquisition				
Vaginal sex is higher risk	124 (37.5)	41.7 (36.4–47.2)	80 (24.5)	27.2 (22.4–32.5)
Anal sex is higher risk	39 (11.7)	7.6 (5.6–10.3)	74 (22.6)	25.0 (20.4–30.2)
Oral sex is higher risk	15 (4.5)	4.0 (2.5–6.5)	9 (2.8)	1.4 (0.7–2.8)
Vaginal, anal and oral sex have similar risk	151 (45.4)	46.7 (41.4–52.1)	161 (49.2)	46.4 (41.0–51.9)
Don’t know	4 (1.2)	1.1 (0.4–2.9)	3 (0.9)	0.9 (0.3–2.9)
Total	333		327	
Knowledge of the type of anal sex with the highest risk of HIV acquisition				
Receptive anal sex is higher risk	57 (17.3)	15.2 (11.8–19.2)	41 (12.5)	16.1 (12.1–21.1)
Insertive anal sex is higher risk	40 (12.1)	12.0 (8.9–16.0)	34 (10.4)	16.0 (11.7–21.4)
Receptive and insertive anal intercourse have similar risk	228 (69.1)	72.8 (67.9–77.3)	222 (67.7)	67.9 (61.9–73.4)
Don’t know	5 (1.5)	1.5 (0.6–3.6)	31 (9.5)	9.6 (6.8–13.4)
Total	330		328	
Concurrent sexual partners during the last 12 months				
Male and female sexual partners				
No	181 (54.5)		175 (53.7)	
Yes	151 (45.5)	45.4 (40.1–50.7)	151 (46.3)	51.8 (46.3–57.2)
Total	332		326	
At least 2 male sexual partners				
No	103 (31.0)		129 (39.6)	
Yes	229 (69.0)	69.3 (64.2–74.0)	197 (60.4)	56.3 (50.8–61.7)
Total	332		326	
At least 2 female sexual partners				
No	221 (66.8)		259 (79.5)	
Yes	110 (33.2)	35.4 (30.4–40.8)	67 (20.6)	24.2 (19.6–29.5)
Total	331		326	
Condom use during the last sexual intercourse				
With a regular male sexual partner				
No	54 (18.1)		82 (28.7)	
Yes	244 (81.9)	79.9 (74.9–84.1)	204 (71.3)	72.5 (67.1–77.3)
Total	298		286	
With a casual male sexual partner				
No	34 (13.0)	85.5 (80.5–89.4)	33 (14.7)	74.5 (66.8–80.9)
Yes	228 (87.0)		191 (85.3)	
Total	262		224	
With a regular female sexual partner				
No	34 (23.0)		27 (20.6)	
Yes	114 (77.0)	83.3 (77.4–87.9)	104 (79.4)	86.3 (80.5–90.7)
Total	148		131	
With a casual female sexual partner				
No	13 (9.2)		11 (10.9)	
Yes	128 (90.8)	94.9 (91.4–97.0)	90 (89.1)	89.6 (82.0–94.2)
Total	141		101	
Consistent use of condoms				
With a regular male sexual partner				
No	143 (48.0)		137 (48.4)	
Yes	155 (52.0)	58.0 (50.2–61.2)	146 (51.6)	57.4 (51.6–63.0)
Total	298		283	
With a casual male sexual partner				
No	79 (30.0)		86 (38.9)	
Yes	184 (70.0)	68.0 (62.1–73.4)	135 (61.1)	56.1 (49.4–62.7)
Total	263		221	
With a regular female sexual partner				
No	74 (50.0)		47 (36.2)	
Yes	74 (50.0)	56.9 (48.9–64.6)	83 (63.8)	68.1 (59.7–75.4)
Total	148		130	
With a casual female sexual partner				
No	34 (24.1)		27 (26.5)	
Yes	107 (75.9)	68.5 (59.9–76.1)	75 (73.5)	73.5 (64.0–81.3)
Total	141		102	
Ever experienced condom breakage during the last 12 months				
No	231 (70.4)	70.8 (65.6–75.5)	221 (67.6)	68.0 (62.7–72.8)
Yes	97 (29.6)	29.2 (24.5–34.4)	106 (32.4)	32.0 (27.2–37.3)
Total	328		327	

In the past 12 months, 45.4 and 51.8% of the participants in Ouagadougou and Bobo-Dioulasso respectively reported they had one male sexual partner and one female sexual partner. The proportion who had at least two male sexual partners was 69.3 and 56.3% in Ouagadougou and Bobo-Dioulasso respectively, whereas 35.4% (Ouagadougou) and 24.2% (Bobo-Dioulasso) reported that they had at least two female sexual partners in the same time period.

Condom use at last sex with a regular male sexual partner was reported by 79.9% of MSM in Ouagadougou and 72.5% in Bobo-Dioulasso, whereas 85.5% of the respondents in Ouagadougou and 74.5% in Bobo-Dioulasso said that they used a condom at last sex with a casual male partner.

83.3% of MSM in Ouagadougou and 86.3% in Bobo-Dioulasso used a condom at last sex with a regular female sexual partner. Condom use at last sex was higher with casual female partners (94.9% in Ouagadougou and 89.6% in Bobo-Dioulasso).

### HIV prevalence

The unadjusted prevalence of HIV among MSM in our sample was 3.6% overall, 3.3% in Ouagadougou and 4.0% in Bobo-Dioulasso (*p* = 0.510). The RDS-adjusted prevalence was 1.9% (95% CI: 1.1–3.5) in Ouagadougou and 2.3% (95% CI: 1.3–4.0) in Bobo-Dioulasso.

HIV prevalence among MSM under 25 years old was 1.3% (95% CI: 0.6–2.8) and 0.9% (95% CI: 0.4–2.5) respectively in Ouagadougou and Bobo-Dioulasso, compared to 5.4% (95% CI: 2.2–12.5) and 6.6% (95% CI: 3.4–12.3) among those over 25 years old in these cities (*p* = 0.010 and *p* < 0.001). MSM with no education or primary school education were more likely to test positive for HIV in Bobo-Dioulasso (7.8, 95% CI: 3.2–17.9) than those with a higher level of education (1.8, 95% CI: 0.4–7.0 for university level and 1.6, 95% CI: 0.7–3.5 for secondary school level, *p* = 0.009). Additionally, in Bobo-Dioulasso informal, public and private sector employees were more likely to test positive for HIV (6.4, 95% CI: 3.6–11.1) than students and pupils (0.3, 95% CI: 0.0–2.2). The HIV prevalence was 11.0%, (95% CI: 3.4–30.2) and 16.1% (95% CI: 4.9–41.7) among MSM who had ever been married to a woman compared to 1.5% (95% CI: 0.7–3.0) and 1.9% (95% CI: 1.0–3.4) among their single counterparts respectively in Ouagadougou (*p* < 0.01) and Bobo-Dioulasso (*p* < 0.001) (Table 3). Significant differences were also observed between HIV prevalence among MSM who had at least one biological child and those who did not in both Ouagadougou (7.3, 95% CI: 2.3–20.9 versus 1.5, 95% CI: 0.8–3.0, *p* = 0.012) and Bobo-Dioulasso (15.4, 95% CI: 6.8–31.5 versus 1.3, 95% CI: 0.6–2.8; p < 0.001). In both cities combined, 6.6% of MSM who reported STI testing during the last 12 months tested positive for HIV, whereas 2.8% of those who did not report STI testing tested positive for HIV (*p* = 0.029). No difference in either city in terms of HIV prevalence was observed among gay, bisexual and heterosexual MSM (*p* = 0.905) or between those who practiced receptive and insertive anal intercourse (Table 3).

**Table 3:** HIV seroprevalence by demographic and behavioral characteristics among men who have sex with men consented for HIV testing in Ouagadougou and Bobo-Dioulasso

Variables	Ouagadougou			Bobo-Dioulasso		
	RDS unadjusted % HIV positive	RDS-adjusted % HIV Positive (95% CI)	*P value*	RDS unadjusted % HIV positive	RDS-adjusted % HIV positive (95% CI)	*P value*
Current age (years)						
<=24	2.2	1.3 (0.6–2.8)	*0.010*	1.6	0.9 (0.4–2.5)	*< 0.001*
> = 25	8.9	5.4 (2.2–12.5)		10.8	6.6 (3.4–12.3)	
Total	3.3	1.9 (1.1–3.5)		4.0	2.3 (1.3–4.0)	
Marital status (with a woman)						
Single	2.6	1.5 (0.7–3.0)	*0.010*	3.2	1.9 (1.0–3.4)	*< 0.001*
Other^a^	15.8	11.0 (3.4–30.2)		25.0	16.1 (4.9–41.7)	
Country of birth						
Burkina Faso	3.3	1.9 (1.0–3.7)	*0.924*	3.6	2.1 (1.1–3.9)	*0.508*
Other countries	3.5	2.1 (0.5–8.0)		5.6	3.3 (1.0–9.9)	
Childhood environment						
Urban	3.4	2.0 (1.1–3.7)	*0.942*	3.6	2.1 (1.2–3.8)	*0.152*
Rural	3.7	2.2 (0.3–14.2)		10.0	6.0 (1.5–21.8)	
Highest educational level						
None or primary	4.0	2.3 (0.3–15.2)	*0.348*	12.8	7.8 (3.2–17.9)	*0.010*
Secondary	2.5	1.4 (0.6–3.2)		2.7	1.6 (0.7–3.5)	
University	6.1	3.6 (1.3–9.3)		3.1	1.8 (0.4–7.0)	
Occupation						
Student/pupil	2.1	1.2 (0.5–3.0)	*0.132*	0.6	0.3 (0.0–2.2)	*< 0.001*
Employed	6.8	4.1 (1.7–9.6)		9.3	6.4 (3.6–11.1)	
Unemployed	4.5	2.7 (0.4–17.1)		0.0	–	
Number of biological children						
0	2.6	1.5 (0.8–3.0)	*0.012*	2.3	1.3 (0.6–2.8)	*< 0.001*
> = 1	12.0	7.3 (2.3–20.9)		24.0	15.4 (6.8–31.5)	
Gender identity						
Man	2.5	1.5 (0.7–3.3)	*0.699*	4.5	2.6 (1.4–5.0)	*0.692*
Woman	4.6	2.7 (0.4–17.1)		4.0	2.3 (0.7–7.1)	
Intersex	5.5	3.2 (1.2–8.6)		1.9	1.1 (0.2–7.5)	
Sexual orientation						
Gay/homosexual	3.5	2.1 (0.9–4.6)	*0.925*	3.8	2.2 (1.1–4.7)	*0.906*
Bisexual	3.4	2.0 (0.8–4.7)		3.9	2.3 (0.9–5.4)	
Heterosexual	0.0	–		7.7	4.6 (0.6–27.2)	
Transgender	0.0	–		0.0		
Had insertive anal sex in the past 12 months						
No	5.3	3.1 (0.4–19.5)	*0.623*	7.6	4.5 (1.9–10.6)	*0.092*
Yes	3.2	1.9 (1.0–3.5)		3.0	1.8 (0.9–3.5)	
Had receptive anal sex in the past 12 months						
No	2.7	1.5 (0.5–04.7)	*0.636*	2.5	1.5 (0.5–4.5)	*0.328*
Yes	3.6	2.1 (1.1–4.2)		4.7	2.8 (1.5–5.2)	
Number of male anal sex partners during the last 12 months						
1	2.7	1.6 (0.4–6.1)	*0.845*	2.2	1.3 (0.3–4.9)	*0.572*
2	3.0	1.8 (0.7–4.6)		4.8	2.8 (1.1–6.4)	
> = 3	4.0	2.4 (1.0–5.6)		4.6	2.7 (1.1–6.5)	
At least 2 sexual partners currently						
No	8.3	1.3 (0.6–2.9)	*0.017*	4.0	2.3 (1.3–4.3)	*0.974*
Yes	2.2	5.0 (2.0–11.6)		4.0	2.3 (0.7–7.0)	
Tested for STIs during the last 12 months						
No	2.5	1.4 (0.6–3.2)	*0.432*	3.1	1.8 (0.9–3.6)	*0.099*
Yes	5.7	3.4 (1.4–8.0)		7.5	4.4 (1.8–10.4)	
Knowledge of the type of sex with the highest risk of HIV acquisition						
Vaginal sex is higher risk	1.6	0.9 (0.2–3.7)	*0.046*	1.3	0.7 (0.1–5.0)	*0.103*
Anal sex is higher risk	7.7	4.6 (1.5–13.6)		0.0	0.0	
Oral sex is higher risk	0.0	0.0		1.1	6.7 (0.9–36.8)	
Vaginal, anal and oral sex have similar risk	3.3	1.9 (0.8–4.6)		6.8	4.1 (2.2–7.3)	
Don’t know	25.0	16.1 (1.9–65.2)		0.0	0.0	
Knowledge of the type of anal sex with the highest risk of HIV acquisition						
Receptive anal sex is higher risk	7.0	4.2 (1.5–10.8)	*0.301*	2.4	1.7 (0.2–11.4)	*0.555*
Insertive anal sex is higher risk	0.0	0.0		2.9	1.4 (0.2–9.6)	
Receptive and insertive anal intercourse have similar risk	2.6	1.5 (0.7–3.4)		5.0	2.9 (1.6–5.2)	
Don’t know	0.0	0		0.0	0	
Concurrent sexual partners during the last 12 months						
Male and female sexual partners						
No	5.0	2.9 (1.5–5.6)	*0.065*	3.4	2.0 (0.9–4.4)	*0.579*
Yes	1.3	0.8 (0.2–3.1)		4.6	2.7 (1.3–5.7)	
At least 2 male sexual partners						
No	2.9	1.7 (0.5–5.2)	*0.785*	3.1	1.8 (0.7–4.8)	*0.509*
Yes	3.5	2.0 (1.0–4.1)		4.6	2.7 (1.4–5.1)	
At least 2 female sexual partners						
No	4.1	2.4 (1.2–4.6)	*0.282*	4.3	2.5 (1.4–4.5)	*0.639*
Yes	1.8	1.1 (0.3–4.2)		3.0	1.7 (0.4–6.8)	
Condom use during the last sexual intercourse						
With a regular male sexual partner						
No	1.9	1.1 (0.1–7.4)	*0.499*	2.4	1.4 (0.4–5.6)	*0.665*
Yes	3.7	2.2 (1.1–4.1)		3.4	2.0 (1.0–4.2)	
With a casual male sexual partner						
No	2.9	1.7 (0.2–11.5)	*0.776*	3.0	1.8 (0.2–11.8)	*0.590*
Yes	4.0	2.3 (1.2–4.4)		5.2	3.1 (1.7–5.7)	
With a regular female sexual partner						
No	5.9	3.5 (0.8–13.3)	*0.195*	7.4	4.4 (1.1–16.6)	*0.433*
Yes	1.8	1.0 (0.2–4.1)		3.9	2.3 (0.8–6.0)	
With a casual female sexual partner						
No	0.0	0.0	*0.661*	0.0	0	*0.506*
Yes	1.6	0.9 (0.2–3.6)		4.4	2.6 (1.0–6.9)	
Consistent use of condoms						
With a regular male sexual partner						
No	4.9	2.9 (1.4–6.0)	*0.158*	2.2	1.3 (0.4–3.9)	*0.359*
Yes	1.9	1.1 (0.4–3.5)		4.1	2.4 (1.1–5.3)	
With a casual male sexual partner						
No	2.5	1.5 (0.4–5.8)	*0.481*	5.8	3.4 (1.4–8.1)	*0.649*
Yes	4.4	2.6 (1.3–5.1)		4.4	2.6 (1.2–5.8)	
With a regular female sexual partner						
No	4.1	2.4 (0.8–7.3)	*0.313*	6.4	3.8 (1.2–11.4)	*0.472*
Yes	1.4	0.8 (0.1–5.5)		3.6	2.1 (0.7–6.5)	
With a casual female sexual partner						
No	0.0	0 .0		0.0	0.0	*0.241*
Yes	1.9	1.1 (0.3–4.3)		5.3	3.1 (1.2–8.3)	
Ever experienced condom breakage during anal sex with men (the last 12 months)						
No	1.7	1.0 (0.4–2.7)	*0.033*	2.7	1.6 (0.7–3.5)	*0.093*
Yes	6.2	3.7 (1.6–8.0)		6.6	03.9 (1.9–8.1)	

^*^Married or cohabiting, divorced, separated

## Discussion

Our study, the first of its kind in Burkina Faso, estimated HIV prevalence among MSM using RDS in the two largest cities of Burkina Faso (Ouagadougou and Bobo-Dioulasso). The study provides useful evidence for HIV epidemic prevention and monitoring programs. The majority of the participants were young MSM with a secondary school level of education or higher, and they were unmarried. Concerning sexual orientation, many reported they were gay and a relatively significant proportion was bisexual. Other studies in Africa have observed similar characteristics relating to youth, sexual orientation and education level of participants [18, 20, 28, 29].

The study showed that HIV prevalence among MSM in Burkina Faso was relatively high compared with the prevalence in the general population, which was 1.0% [22]. Specifically, the prevalence among young men between the ages of 15–20 years and 20–24 years in the general population, were respectively 0.4 and 0.5% at the national level [22]. These data confirm the higher prevalence of HIV among key populations such as MSM in countries with generalized epidemics [6, 11].

Our study found a higher HIV prevalence among MSM in Burkina Faso compared to a 2011 study in Nigeria where HIV prevalence was found to be 1.1% among MSM [30]. However, the HIV prevalence among MSM found in our study is one of the lowest in Sub-Saharan Africa [17, 18, 28]. The low HIV prevalence may be explained by the fact the sample mainly includes young people who do not have significant cumulative risk. A stratification by age group showed a higher prevalence among MSM at least 25 years old in Ouagadougou (5.4%) and in Bobo-Dioulasso (6.6%). Other studies in Sub-Saharan Africa have found higher HIV rates among older MSM compared to this study. In Uganda, while HIV prevalence among young male adults was 4.5%, a study showed a 22.4% prevalence among MSM who were at least 25 years old, significantly different from the prevalence among 18–24 year old MSM, which was 3.9% [31]. Similar differences between age groups were observed in the Gambia [32].

The high HIV prevalence among MSM in Sub-Saharan Africa may be because it is the geographic region with the highest HIV prevalence. However structural factors including stigmatization and criminalization of same-sex sexual practices may also make MSM more vulnerable to HIV infection and limit their access to prevention and care [2, 14, 33]. Estimations made according to the Modes of Transmission model of UNAIDS showed prevalences of 16.8–40% in Southern Africa, 18–43% in East Africa 10–25% in West Africa [34]. In a recent systematic review, the authors estimated the prevalence at 17.7% among MSM in central and West Africa [35]. These high rates of prevalence highlight the need for targeted actions in the response to the pandemic in Sub-Saharan Africa.

Many MSM surveyed in this study had sexual intercourse with at least one male and one female sexual partner during the last 12 months. Among more than half of MSM, partnerships with multiple male partners or multiple female partners were observed. Most of the studies in Sub-Saharan Africa have reported inconsistent use of condoms among MSM [13, 15–17]. A large proportion of MSM (82.9%) were not aware of the specific risks of anal sexual intercourse compared with the vaginal intercourse. They are also not aware of the risk difference between insertive and receptive anal intercourse (85.1%). Studies have reported that the risk of HIV transmission is about 20 times higher through receptive anal sexual intercourse than vaginal sexual intercourse, which implies a higher risk of infection among MSM [36, 37]. Many MSM do not have specific knowledge on the risk of HIV infection and sexual intercourse between men. This is significant in several African countries where awareness raising and HIV prevention programs focus on heterosexual transmission and mother to child transmission of HIV. These programs have not included information on sexual transmission between men, which is still considered taboo and condemned by society [33, 38]. To change this lack of knowledge, HIV program may spread safe sexuality education, among MSM and appropriate language in HIV awareness campaigns.

It is reported that in the countries where homosexuality is considered as a crime, the implementation of HIV programs intended for MSM is difficult, and their access to health centers is compromised [8, 14, 39]. In Burkina Faso, although same sex practices are stigmatized by the society, they are not punished by the law. This enables prevention programs to undertake public health actions for MSM in order to control the spread of HIV. Our study shows that MSM under 25 years old are still less likely to be living with HIV. Those young MSM should serve as a target to raise awareness and promote safer sex to prevent HIV acquisition. We tried to start older seeds who were then unable to recruit. The low representation of older MSM in our study, likely due to fear of being stigmatized and discriminated against, calls for innovative strategies for larger HIV prevention interventions coverage for all age groups. These programs should focus on educating on safer behaviors, promoting condom use, prevention and treatment of STIs and HIV, fighting against discrimination and stigmatization, and improving access to health centers [5, 10, 14, 40]. These interventions are critical because unsafe behaviors were observed among MSM, and some of them were not aware of the risks.

One of the weaknesses of our study is the low inclusion of older MSM, among whom HIV prevalence is higher because of the accumulation of risks. Another limitation to be considered in this study was the lack of multivariate analyses to assess the association between HIV status and participant’s sociodemographics and behavior characteristics in each city due to the low of HIV positive number.

Self-reported data are also subject to inaccurate recall and social desirability bias, particularly when reporting sexual behaviors in a context where homosexuality is stigmatized. Finally, there are weaknesses relating to the use of the RDS method during epidemiological studies [41]. However, RDS is an innovative and efficient strategy that is used for the recruitment of MSM [42], particularly because of the limited physical and online venues in which MSM meet in Burkina Faso. Despite these limitations, our study is able to provide data on HIV prevalence among MSM in Burkina Faso to complement the existing data available in Sub-Saharan Africa for HIV control programs.

## Conclusions

This study showed a high HIV prevalence among MSM in Burkina Faso compared to the general population, but it remains low compared with data reported among MSM in several African countries. However, this low HIV prevalence must be interpreted in light of the very young age of our study participants.

The low HIV prevalence among MSM in Burkina Faso is an opportunity for HIV prevention and a challenge for HIV control programs to raise awareness among these populations and promote safer sex, particularly for young people, to mitigate HIV transmission. For this purpose, these programs need support from donors for innovative actions such as promoting and providing pre-exposure prophylaxis, condoms and water-based lubricants, HIV counseling, testing, early treatment initiation and effective involvement of the MSM communities in the response to the HIV epidemic.

## Abbreviations

CERS: Comité d’éthique pour la recherche en santé
CI: Confidence interval
HIV: Human Immunodeficiency Virus
IRSS: Institut de Recherche en Sciences de la Santé
MSM: Men who have sex with men
RDS: Respondent driven sampling
STI: Sexually transmitted infection
USD: United States dollars
XOF: West African Communauté Financière franc
